# The Psychological Resilience of Older Adults Is Key to Their Independence in Instrumental Activities of Daily Living and Social Participation

**DOI:** 10.3390/brainsci15040383

**Published:** 2025-04-07

**Authors:** Noelia Saez-Sanz, Encarnacion Sanchez-Lara, Raquel Gonzalez-Perez, Alfonso Caracuel, Isabel Peralta-Ramirez

**Affiliations:** 1 Department of Developmental and Educational Psychology, University of Valladolid, 47002 Soria, Spain; noelia.saez@uva.es; 2 Mind, Brain, and Behavior Research Center (CIMCYC), University of Granada, 18071 Granada, Spain; acaracuel@ugr.es (A.C.); mperalta@ugr.es (I.P.-R.); 3 Department of Personality, Assessment, and Psychological Treatment, University of Granada, 18071 Granada, Spain; 4 Department of Pharmacology, Centro de Investigación Biomédica en Red de Enfermedades Hepáticas y Digestivas (CIBERehd), School of Pharmacy, University of Granada, 18071 Granada, Spain; raquel.gonzalez@ciberehd.org; 5 Department of Developmental and Educational Psychology, University of Granada, 18071 Granada, Spain

**Keywords:** instrumental activities of daily living, participation, resilience, hair cortisol, older adults

## Abstract

**Background/Objectives**: The link between stress and performance in instrumental activities of daily living (IADLs) and participation in older adults is gaining importance. The existing evidence is based on single measures of salivary cortisol levels; therefore, there is a need for more comprehensive studies that incorporate long-term measurements of cortisol concentrations as indicators of chronic stress. In consequence, the objective is to determine whether perceived stress, hair cortisol concentration, and psychological resilience are related to IADLs and participation in older individuals. **Methods**: A sample of 63 individuals with a mean age of 76.5 years underwent an assessment of stress variables (Perceived Stress Scale, hair cortisol concentration, and Resilience Scale), IADLs (UPSA Scale), and participation (PART-O Scale). Using the stress variables as factors, multiple linear regressions were conducted to predict UPSA and PART-O scores and their respective subscales. The correlation between UPSA and PART-O was also examined. **Results**: After controlling for age, gender, and cognitive status, resilience emerged as the sole independent predictor of overall scores on both scales, as well as on two subscales: UPSA-Communication and PART-O-Others, for which hair cortisol was also a predictor. The effect size of the association between UPSA and PART-O scores was small. **Conclusions**: psychological resilience is not only a protective variable against stress but also appears to be associated with instrumental functioning and social participation in older adults. This finding suggests that resilience plays a role in facilitating IADLs and participation among the elderly population.

## 1. Introduction

Population aging is a global phenomenon projected to increase the proportion of individuals aged 65 and over to 16% by 2050 [1]. This demographic shift poses significant financial challenges for old-age support systems worldwide, primarily due to the high levels of dependency among older adults [2]. Dependency occurs when individuals cannot perform Activities of Daily Living (ADLs) autonomously despite environmental adaptations or the use of technical aids [3]. One of the World Health Organization’s (WHO) recommendations to address dependence is the promotion of participation, given its association with factors such as successful aging [4], reduced risk of decline in ADLs, and the need for long-term care [5]. According to the WHO International Classification of Functioning (ICF), participation is defined as “the act of engaging in a life situation,” while activity is referred to as “the performance of a task or action by an individual” [6]. Other authors define participation as the set of activities undertaken by an individual to fulfill their social roles as a member of society [7]. This definition incorporates the social perspective of participation [8] and allows for a clear distinction between activity and participation, which is not explicitly stated in ICF [9].

Given that both independence for Instrumental Activities of Daily Living (IADLs) and participation have been associated with successful aging [4,5], it is crucial to study the protective and risk factors of both constructs. Protective factors related to IADL performance include social integration, residing in rural areas, engaging in high levels of physical activity, and possessing resilience [10,11,12]. On the other hand, there is a wide range of risk factors, such as being female, being older, having a limited education, having low income, being separated or divorced, living with people other than a spouse, and suffering from depressive symptomatology, cognitive impairment, and chronic illnesses [12,13,14]. Additionally, some findings suggest a relationship between stress and decreased independence in IADLs. Feng et al. [15] reported that older individuals, especially those over 75 years, experienced heightened psychological stress when their functional abilities were impaired, highlighting the role of perceived social support in buffering such stress.

In terms of participation, several protective factors have been identified. These include motivation, neighborhood cohesion, and a high level of social support [16], along with psychological resilience [10,17]. Risk factors include age, particularly being over 75 years old [18]; low educational and socioeconomic status; poorer cognitive, physical, and mental health management function; difficulties in accessing social activities [16,19]; and stress [20]. Regarding the relationship between participation and stress, Tomioka et al. [20] have built upon the findings of other authors, such as Cohen [21], by hypothesizing that socially participative adults have much more frequent interactions with others and a more positive emotional outlook, which could contribute to lower levels of stress.

While stress appears to be a common risk factor for maintaining independence for IADLs and participation, the relationships between these constructs remain relatively understudied. However, some findings suggest a connection between perceived stress and decreased functionality for these activities in some populations. For instance, studies have shown that perceived stress is related to decreased independence in performing IADLs in adults with autism [22] and with physical disability in people with arthritis aged 60 years or older [23].

Stress triggers significant physiological changes by activating the pituitary–pituitary–adrenal (HPA) axis, increasing cortisol secretion [24]. Several studies have examined the association between salivary cortisol levels level and ADL performance. For instance, Wrosch et al. [25] found an association between salivary cortisol and dependence in both ADLs and IADLs in people aged over 60 years, a relationship that was mediated by health-related control strategies, while Shindel et al. [26] found a similar relationship in older adults with depression. A later study also found a link between salivary cortisol dysregulation and poorer functionality, although engaging in volunteer activities had a beneficial moderating effect [27]. Similarly, Zilioli et al. [28] reported a negative correlation between engaging in social activities and salivary cortisol levels in older African Americans. To date, we have only found studies that relate functionality to cortisol measured in saliva, which provides only a snapshot of cortisol levels at a specific moment rather than a stable measure of chronic stress. In contrast, studying cortisol concentrations in hair allows for assessing chronic hypothalamic–pituitary axis activation in the past three months, providing a more stable measure of cortisol fluctuations [29,30].

Resilience is another important variable to consider in studying IADL performance and participation [10]. Psychological resilience refers to the process by which a person adequately adapts to or recovers from adversity, trauma, tragedy, threat, or significant sources of stress [31]. Greater resilience is associated with higher performance on IADLs and better health-related quality of life in people with mild cognitive impairment [32]. In addition, higher levels of resilience in older people, especially those aged 65–85 years, are predictive of a lower risk of dependence on ADLs [33]. This association has also been observed in adults with HIV aged between 40 and 73 years [34,35]. In addition, another recent longitudinal study has found a relationship between psychological resilience and long-term IADLs [36]. Regarding the relationship between resilience and participation, studies have demonstrated a positive association between these constructs in people aged over 60 years [17], which is moderated by the sense of belonging to the community [37].

Finally, several studies have determined the relationship between the constructs of participation and IADL among community-dwelling older adults, finding that social activity is associated with a lower risk of dependency in IADL [5,38].

In summary, while research on the link between stress and psychological resilience and performance in IADLs and participation in older adults is gaining importance, studies remain scarce in this area. Moreover, the existing evidence is based on single measures of salivary cortisol levels, which may not capture chronic stress accurately. Therefore, there is a need for more comprehensive studies that incorporate long-term measurements of cortisol concentrations as indicators of chronic stress. Accordingly, the present study aimed to determine whether perceived stress, hair cortisol concentration, and psychological resilience are related to IADLs and participation in older people, as well as the relationship between the latter two variables.

## 2. Materials and Methods

### 2.1. Participants

The sample consisted of 63 individuals, of whom 68.8% were women. The participants were aged between 58 and 93 years (M = 76.49 years, SD = 8.35), with a mean of 8.7 years (SD = 4.02) of education and a mean MMSE score of 27.30 (SD = 2.42). The inclusion criteria for the study were as follows: (i) being older than 55 years, as this stage marks the onset of cognitive, emotional, and social changes that influence successful aging [39,40,41]; (ii) having a basic level of literacy; (iii) having an MMSE score ≥21, as recommended by MacKenzie et al. [42]; and (iv) not having a diagnosis of dementia. Exclusion criteria include having individuals with corticosteroid treatment, those diagnosed with a psychiatric disorder, individuals with diabetes, or those with a diagnosis of dementia or a major mental disorder.

Since no previous studies closely match ours in terms of population, variable types, and analysis approach, we based our sample size estimation on the study by Wong et al. [43], which showed notable similarities. Their study involved an elderly population with a mean age of 76.5 years and examined variables similar to ours: cognitive functioning, stress, and activities of daily living. They reported large effect sizes for the associations between stress reduction ability and improvements in both cognitive functioning and daily living activities.

To adopt a more conservative approach due to methodological differences between our study and theirs, we used a moderate effect size (f^2^ = 0.25) for our sample size calculation using the G*Power software 3.1.2 [44]. For a linear multiple regression model (F test: fixed model, R^2^ deviation from zero) with six predictors and a statistical power of 0.80, the estimated required sample size was N = 62.

All participants gave informed consent. The study was conducted in accordance with the Declaration of Helsinki [45] and the Good Clinical Practice Directive (Directive 2005/28/EC) of the European Union. The study was approved by the Human Research Ethics Committee of the University of Granada (235/CEIH/2016) on 9 September 2016. The data can be found on the following website: https://osf.io/5pb9a/ (accessed on 20 July 2023).

### 2.2. Instruments

#### 2.2.1. Tests to Assess Cognitive Status

- Mini-Mental State Examination (MMSE) [46]. This is the most widely used instrument to measure global cognitive performance. The Cronbach’s alpha was 0.91 [47]. The global score was used to screen for participation in the study.

- Addenbrooke’s Cognitive Examination 3rd version (ACE-III) [48]. This brief cognitive assessment test includes attention, memory, language, verbal fluency, and visuospatial skills. The global and subscales scores were used in this study. The Cronbach’s alpha was 0.93 [49].

#### 2.2.2. Instrumental Activities of Daily Living Measurement

The UCSD Performance-Based Skills Assessment (UPSA) [50] was adapted from Goldberg et al. [51]. This is a performance-based measure of functional skills with adequate ecological validity. It consists of four subscales: finance (ability to perform tasks related to money exchange, such as shopping and interpreting bills), communication (skills for making phone calls in emergency situations, as well as running errands and handling medical tasks), understanding and planning recreational tasks (ability to plan a trip by organizing how and what they have to take based on information provided in written form), and transportation (use of public transportation using maps and bus schedules). Each subscale is assigned a percentage score, with a maximum value of 25, so that the overall score ranges from 0 to 100. The Cronbach’s alpha in a Spanish sample of people with schizophrenia was 0.90 [52].

#### 2.2.3. Participation Measure

The Participation Assessment with Recombined Tools-Objective (PART-O) [53]. This instrument evaluates the quantity, frequency, and type of activities a person engages in to fulfill their social roles. It consists of four subscales: productivity (work and household chores), social relationships (frequency of socializing with friends or family, emotional support, communication via the internet, having a partner or a close friend), out and about (frequency of activities conducted outside the home, in stores, restaurants, cinema, religious events, and participating in sports or sporting events), and other forms of participation (frequency of engaging DIY tasks, using public transport, or driving one’s own vehicle, volunteering, attending association meetings, participating in training activities, and leaving one’s living space). A high score is taken to indicate a high level of participation. The instrument has shown good reliability, with a coefficient of 0.86 reported for samples of individuals with head injuries.

#### 2.2.4. Stress Measures

Perceived stress: The Perceived Stress Scale [54] (PSS), in its Spanish version, was developed by Remor and Carrobles [55]. This instrument measures how individuals evaluate their lives as stressful in the last month. It consists of 14 items (7 direct and 7 inverse) using a Likert scale from 0 (never) to 4 (very often). The score ranges from 0 to 56, and higher scores are taken to indicate greater perceived stress. A review by Cohen et al. [54] reported a Cronbach’s alpha greater than 0.84–0.86 for various populations.

Resilience: The Connor and Davidson Resilience Scale (CD-RISC) [56]. The score on this instrument ranges from 0 to 100, with higher scores taken to indicate a greater level of resilience. The scale evaluates 5 components: (i) personal competence, measuring levels of demand and tenacity; (ii) trust in one’s own instincts, measuring the ability to tolerance negative affect and effectively cope with stress; (iii) positive acceptance of change and secure relationships; (iv) control; and (v) spiritual influences. We used the overall total score in this study. The original study reported a Cronbach’s alpha of 0.89, while the Spanish adaptation yielded a value of 0.86 [57].

Hair cortisol concentration (HCC): The cortisol evaluation involved cutting a tuft of hair consisting of approximately 150 strands obtained from the back of the skull, as close to the scalp as possible [58]—for each sample, a maximum length of 3 cm was established to capture cortisol levels over the previous three months [59]. The samples were wrapped in aluminum foil to protect them from light and humidity and stored at room temperature until further analysis. This analysis was conducted using a protocol previously published by Romero-Gonzalez et al. [60]. Percentile scores of hair cortisol validated for the Spanish population were used to determine cortisol levels [61].

### 2.3. Procedure

The study participants lived in Granada, and their assessment took place in community centers within the same province. The sample was recruited through distribution lists provided by the Granada City Council, the University of Granada, and the professionals working at the community centers. The assessments were conducted individually by a trained psychologist and lasted approximately three hours in total. The sessions were divided into three parts, with a break in the middle of each session. Following the completion of the final session, a hair cortisol sample was collected. Participants completed a sociodemographic questionnaire and the ACE-III assessment during the first session. The UPSA and the CD-RISC were administered in the second session, and the PSS and PART-O were administered in the third session.

### 2.4. Analysis

Linear regression analyses were conducted to explore the predictive relationship between stress measures and the total scores on each performance subscale of the IADLs and the participation scale. First, to predict the IADLs, the total UPSA score and each of its subscales (finance, communication, planning and understanding, and mobility) were used as dependent variables. Second, to predict participation, the dependent variables were the total score of PART-O and each of its subscales (productivity, social relations, external and other forms of participation). All models included resilience scores, perceived stress, and HCC as independent variables. In addition, three independent variables that showed potential relationships between performing activities of daily living and participation were included in all analyses. These variables were age [14,19] and gender [62,63]. Finally, the Pearson correlation coefficient between UPSA and PART-O scale scores was determined. The data were analyzed using the SPSS Statistics version 28 [64].

## 3. Results

Table 1 shows the descriptive statistics for cognition, performance on the UPSA and PART-O scales, and stress variables included in the regression models.

In the multiple linear regression analyses conducted for the UPSA, all models were significant except for the understanding and planning subscale (see Table 2). Age and the ACE-III overall score were significant predictors. Regarding the stress variables, only resilience emerged as a significant predictor in the overall score and the communication subscale. The full model explained 51.8% of the variance in the UPSA overall score.

For the PART-O scale, the models were significant for the total score and productivity and other forms of participation (Others) subscales. Age was a predictor in all significant models, and gender was only a predictor in the Others subscale. Resilience was a predictor in the overall score and the Others subscale. The full model explained 12.5% of the variance in the PART-O overall score.

In all models, the Durbin–Watson statistic yielded values within the range of 1.5–2.5, thus ensuring the independence of errors [65]. In addition, variance inflation levels (VIF) were conducted to examine collinearity. The VIF score was below 10 in all cases, indicating non-collinearity [66].

Finally, the Pearson correlation coefficient between the PART-O and UPSA scales was 0.250 (*p* = 0.050).

## 4. Discussion

This study aimed to determine the relationship between functionality in instrumental activities of daily living and participation in older adults, along with their perceived stress levels, hair cortisol, and psychological resilience. Age, cognitive status, and gender were included as control variables due to their known impact on IADL performance [14,62,67] and participation [16,19,63]. The results highlight the significance of psychological resilience as a predictor of overall performance in IADLs, particularly in the communication subdomain. Furthermore, resilience emerges as a predictor of overall participation and its other forms of participation subscale.

Regarding performance in IADLs, our findings indicate that the predictive factor for stress is psychological resilience, i.e., how older adults manage stress, as opposed to the level of perceived stress or hair cortisol. Regarding perceived stress, to our knowledge, apart from our study, its relationship with IADLs in healthy older adults has not been explored. However, both variables have been found to be related in samples of elderly populations with pathologies such as arthritis [23] or autism [22], so the relationship could be mediated by such pathologies. Concerning cortisol as a physiological correlate of stress, studies by Shindel et al. [26] and Huo et al. [27] found an association with IADLs that was not found in our study. However, a key difference between these two studies and ours is the type of cortisol measurement utilized. The mentioned studies collected saliva samples, which capture cortisol levels at a specific point in time and can vary depending on the time of sampling [29]. In contrast, we measured hair cortisol concentrations, which is recommended for assessing chronic stress because it provides a more stable representation of an individual’s state over an extended period of 12 weeks [30]. Therefore, it allows us to determine the presence or absence of prolonged physiological stress states.

The results showed that the variable associated with functionality in IADLs is psychological resilience, which is considered a protective factor against stress. Similar findings have been reported in people with human immunodeficiency virus (HIV) aged between 40 and 73 years [34], where the relationship between functionality in IADLs and resilience was explored (as in the present study) while controlling for cognitive performance as a covariate. Another study conducted in China by Yang and Wen [33] found that higher resilience in individuals over the age of 65 years predicted a lower incidence of ADL. The relationship was stronger in individuals under the age of 85, suggesting potential variations in the association across different age cohorts. Concerning possible explanations for this association, a previous study conducted by our group found that resilience is also a predictor of cognitive performance in older individuals [68]. It is plausible that the cognitive abilities associated with resilience could partially explain the relationship between IADLs and resilience. Evidence to support this notion comes from a study conducted through interviews with older individuals, which revealed that those with the highest levels of resilience implemented cognitive capacities such as anticipating potential losses, proactive planning, and decision-making to address challenges. These resilient individuals strategically made choices, such as relocating to a more convenient living arrangement or actively engaging with relatives or neighbors to preserve their independence and access essential services on a daily basis. In addition, these more resilient individuals reported utilizing the knowledge and skills acquired throughout their lives to maintain their current health for as long as possible [69]. When analyzing the results of the UPSA subscales in this study, we observed that psychological resilience was associated with the finance, communication, and mobility subscales but not with the understanding and planning subscale. This may be explained by the very high scores obtained by participants on this subscale (mean of 23.48 out of a maximum of 25), suggesting a limited ability to discriminate between different levels of performance on the measured activities. Similar results have been reported in the cognitively healthy control group assessed in a previous study with a Spanish population, where the mean score on this subscale was 22.9 [52]. These findings highlight the need to revisit the item difficulty or scoring criteria of this subscale in future research.

Our results also indicate that resilience is associated with participation. To our knowledge, the few existing studies on this issue have failed to explain the nature of the relationship between the two constructs, although several authors suggest that resilience is associated with a key aspect of social participation, such as social support [70,71]. Our study, however, found no evidence to suggest that resilience is specifically associated with the social relations subscale of participation. In this regard, Kawachi and Berkman [72] found that, although paradoxical, social relationships can negatively impact mental health when people perceive themselves as being primarily responsible for providing social support to others. This aspect was not addressed in our study and thus may be important to consider in future research.

These findings are consistent with neurobiological evidence suggesting that prolonged exposure to elevated cortisol levels due to chronic stress disrupts the regulation of the hypothalamic–pituitary–adrenal (HPA) axis, affecting brain structures such as the amygdala, hippocampus, and medial prefrontal cortex [73]. As a result of this dysfunction, various cognitive functions—particularly memory, decision-making, and planning—may be impaired [74]. This, in turn, can compromise an individual’s ability to maintain independence in instrumental activities of daily living (IADLs) and to engage in social participation [5,20].

At the neurobiological level, resilience functions as a protective mechanism by modulating the HPA axis response, thereby reducing the impact of stress on cognitive functioning [68] and encouraging the use of adaptive coping strategies in stressful situations [75]. This is associated with greater autonomy in performing IADLs [15] and a higher degree of social participation [17].

In addition, psychosocial factors such as social support play a crucial role in stress regulation. A strong social network not only dampens HPA axis activation during stressful experiences [76] but also supports key cognitive functions, including memory and executive functioning [77]. These benefits, in turn, contribute to enhanced autonomy in IADL performance and greater participation in meaningful activities [5,20].

Regarding the secondary objective of our study, we found a small but significant relationship between the two primary study variables: functionality in IADLs and participation. This finding aligns with two previous studies that focused on older adults’ membership in social groups as a measure of participation [5,38]. However, our study utilized a broader scale to measure participation, considering not only social relationships but also other aspects, such as productivity and activities conducted outside the home. Therefore, it is crucial to continue delving into the potential role of resilience in all areas of participation.

The small effect size observed between IADL performance and participation suggests that, while these are distinct constructs, they share certain elements that may influence the ability to engage in social roles. IADLs may support participation by providing the autonomy necessary for community involvement, although their overall impact appears to be limited. This indicates that, although participation can be incorporated into programs aimed at enhancing IADL performance, such interventions should not focus solely on participation. Likewise, programs designed to foster participation may benefit from including components that strengthen IADL performance to maximize functional autonomy, but they should also address other key factors, such as access to community networks and the promotion of social support. Sustaining IADL performance over time may help maintain participation, but this should be considered part of a broader, multifaceted approach.

While the findings of this study offer valuable insights as an initial approach to exploring the relationship between resilience and functionality in older adults, it is important to acknowledge certain limitations. In addition, the sample size remains a limitation in terms of the generalizability of the findings. The recruitment of participants through community centers may have introduced selection bias, potentially favoring individuals with better health status and greater social engagement. The sample’s gender composition, which is predominantly female (68.8%), may also limit generalizability, as gender differences could influence the relationships between stress, cognitive functioning, and instrumental activities of daily living (IADLs). Moreover, the broad age range of participants (58–93 years) represents another potential limitation, as associations among the studied variables may differ across age cohorts. Future studies should aim to include more gender-balanced samples and address age range variability with larger and more representative populations to better understand how these relationships may vary across different demographic groups. From future perspectives, longitudinal studies could also be of value in determining the potential capacity of resilience and hair cortisol as stress-related predictors in the evolution of functionality in IADLs and participation. Similarly, a recent longitudinal study proposes a model in which resilient older adults, when facing a problem, draw on their life experience to reduce coping effort, preserving their resources without compromising their effectiveness [78]. It would be relevant to analyze the relationship between psychological resilience, the progression of instrumental activities of daily living (IADLs), and their level of participation. Finally, although the PART-O is a widely used instrument, its focus is exclusively objective and does not capture participants’ subjective experiences. Future studies could benefit from incorporating measures that assess both satisfaction and the perceived importance of participation, offering a more comprehensive understanding of its impact on the emotional well-being of older adults.

The findings of the study have potential implications for social and healthcare interventions. The relationships found could inform the design of interventions that promote psychological resilience, participation, and maintenance of the functional capacity to carry out IADLs. Furthermore, understanding the relationship between stress, coping mechanisms, participation, and functionality can guide the development of intervention strategies to favor successful aging. This is particularly significant, considering that resilience has been found to be associated with participation and functionality [79]. Therefore, such synergistic approaches could improve the effectiveness of programs aimed at promoting successful aging, aligning with the WHO’s campaign for healthy aging in the 2020–2030 decade [80].

## 5. Conclusions

In conclusion, after controlling for age, gender, and cognitive status, psychological resilience emerges as the stress-related factor that best predicts functionality in IADLs and participation among individuals aged 55 and above. Additionally, hair cortisol concentration is also associated with certain forms of participation. Finally, it is important to note that while the association between IADL performance and participation is statistically significant, its effect size is relatively small.

## Figures and Tables

**Table 1:** Descriptive statistics of cognition, UPSA, PART-O, and stress variables.

Test	Variable	Mean	SD	Range
UPSA	Overall score	76.36	17.88	16.25–100
PART-O	Raw overall score	36.35	12.06	16–65
ACE-III	Raw overall score	76.97	11.86	48–97
	Percentil	38.06	24.45	2–97
CD-RISC	Raw overall score	69.78	17.48	17–97
PSS	Raw overall score	18.25	10.50	2–50
Hair Cortisol	Raw score (pg/mg)	129.65	172.56	3.39–675.6
	Percentil	39.35	34.57	0.2–100

Note. UPSA: The UCSD Performance-Based Skills Assessment; PART-O: The Participation Assessment with Recombined Tools-Objective; ACE-III: Addenbrooke’s Cognitive Examination 3rd version; CD-RISC: The Connor–Davidson Resilience Scale; PSS: The Perceived Stress Scale.

**Table 2:** Results of the multiple regression models.

Dependent Variable	Age p	Gender p	ACE-III p	PSS p	HCC p	CD-Risc p	Full model Adj R^2^ (p)	Significant Contributors (St β)
**UPSA**								
Overall Score	<0.001	0.362	<0.001	0.289	0.400	0.037	0.518 (<0.001)	Age (−0.469) ACE-III (0.439) CD-Risc (0.232)
Finance	<0.001	0.093	0.001	0.818	0.234	0.093	0.451 (<0.001)	Age (−0.439) ACE-III (0.368)
Communication	<0.001	0.577	0.002	0.292	0.861	0.036	0.419 (<0.001)	Age (−0.498) ACE-III (0.330) CD-Risc (0.256)
Understanding and Planning	0.256	0.787	0.048	0.371	0.982	0.588	0.026 (0.286)	
Mobility	0.001	0.356	<0.001	0.540	0.319	0.164	0.387 (<0.001)	Age (−0.372) ACE-III (0.425)
**PART-O**								
Overall Score	0.006	0.358	0.994	0.117	0.103	0.030	0.125 (0.034)	Age (−0.362) CD-Risc (0.324)
Productivity	<0.001	0.084	0.199	0.391	0.404	0.638	0.443 (<0.001)	Age (−0.621)
Social Relations	0.758	0.889	0.378	0.377	0.094	0.240	−0.004 (0.558)	
Out and About	0.195	0.699	0.560	0.321	0.714	0.132	−0.038 (0.715)	
Other forms of participation	<0.001	0.019	0.823	0.132	0.032	0.025	0.251 (0.001)	Age (−0.479) Gender (0.285) Hair Cortisol (−0.243) CD-Risc (0.308)

Note. ACE-III: Addenbrooke’s Cognitive Examination 3rd Version; PSS: The Perceived Stress Scale; HCC: Hair cortisol concentration; CD-RISC: The Connor–Davidson Resilience Scale; UPSA: The UCSD Performance-Based Skills Assessment; PART-O: The Participation Assessment with Recombined Tools-Objective. Adj R^2^: Adjusted R squared. St β: Standardized β coefficients.

## Data Availability

https://osf.io/5pb9a/ (accessed on 20 July 2023).
